# Increasing fMRI Sampling Rate Improves Granger Causality Estimates

**DOI:** 10.1371/journal.pone.0100319

**Published:** 2014-06-26

**Authors:** Fa-Hsuan Lin, Jyrki Ahveninen, Tommi Raij, Thomas Witzel, Ying-Hua Chu, Iiro P. Jääskeläinen, Kevin Wen-Kai Tsai, Wen-Jui Kuo, John W. Belliveau

**Affiliations:** 1 Institute of Biomedical Engineering, National Taiwan University, Taipei, Taiwan; 2 Department of Biomedical Engineering and Computational Science, Aalto University, Espoo, Finland; 3 Athinoula A. Martinos Center for Biomedical Imaging, Massachusetts General Hospital, Harvard Medical School, Boston, Massachusetts, United States of America; 4 Institute of Neuroscience, National Yang-Ming University, Taipei, Taiwan; Universiteit Gent, Belgium

## Abstract

Estimation of causal interactions between brain areas is necessary for elucidating large-scale functional brain networks underlying behavior and cognition. Granger causality analysis of time series data can quantitatively estimate directional information flow between brain regions. Here, we show that such estimates are significantly improved when the temporal sampling rate of functional magnetic resonance imaging (fMRI) is increased 20-fold. Specifically, healthy volunteers performed a simple visuomotor task during blood oxygenation level dependent (BOLD) contrast based whole-head inverse imaging (InI). Granger causality analysis based on raw InI BOLD data sampled at 100-ms resolution detected the expected causal relations, whereas when the data were downsampled to the temporal resolution of 2 s typically used in echo-planar fMRI, the causality could not be detected. An additional control analysis, in which we SINC interpolated additional data points to the downsampled time series at 0.1-s intervals, confirmed that the improvements achieved with the real InI data were not explainable by the increased time-series length alone. We therefore conclude that the high-temporal resolution of InI improves the Granger causality connectivity analysis of the human brain.

## Introduction

Determining causal mechanisms by which different brain areas interact to support cognition and behavior has been a persistent challenge in neuroscience. Whereas analyzing synchronization of cerebral activations can identify cortical areas acting in concert, revealing causal influences among them requires measures of *effective connectivity* [1]–[3]. Previously, effective connectivity analyses of human PET [4] and fMRI [5]–[8] data have been conducted using structural equation modeling (SEM), which aims at determining directional modulations across the activated areas using covariance or correlation matrices derived from the measured time series [9]. However, a major limitation of SEM is that it requires strong *a priori* assumptions on the number and directionality of connections, which are often difficult to justify or validate. Similar limitations exist in dynamic causal modeling (DCM), which also requires *a priori* models of directional connections [10]–[12]. To circumvent such limitations, the technique of Granger causality [13] has been applied to data obtained with both EEG [14]–[22] and fMRI [18], [23]–[29]. The main advantage of Granger causality over SEM and DCM is that it can estimate the directionality of modulations within a network without *a priori* assumptions on which connections are active and on directions of the connections. Essentially, Granger causality tests how additional information improves prediction of the future of a given time series. In other words, a Granger causal influence from a time series “X” to time-series “Y” exists if the combined information from “X” and “Y” predicts the future of “Y” better than information from “Y” alone.

Functional MRI of the human brain [30] with blood oxygenation level dependent (BOLD) contrast [31], [32] is the prevailing method for studying brain functions noninvasively. There are two major limitations to using BOLD fMRI for causality modelling. First, BOLD signals are vascular responses that lag the underlying neuronal events by seconds [33] and may show notable voxel-to-voxel latency variability at the individual level [34]. However, it has been suggested that with appropriate modelling to obtain neuronal activity estimates, BOLD fMRI can still be used for causality modelling [35]. The other challenge for using BOLD fMRI in Granger causality estimation is the rather low sampling rate, which is critically important in all time series modeling. Typically fMRI Granger causality analyses use data sampled at the rate of approximately 1–2 s [24], [26]–[29]. Such a slow sampling rate, which is necessary to achieve whole-brain fMRI coverage at a spatial resolution of 3–4 mm, provides only about 10–15 samples during the 20–30 sec duration of a canonical hemodynamic response function [36]. Estimating Granger causality from fMRI time series recorded at such a low sampling rate can be problematic.

Using the recently developed dynamic functional magnetic resonance inverse imaging (InI), one can achieve an order of magnitude faster sampling rate. InI is based on the utilization of simultaneous measurements from multiple channels of an RF head coil array, and by solving sets of inverse problems InI can detect dynamic changes of the BOLD fMRI signals at ∼10 Hz sampling rate with whole-brain coverage and approximately 5-mm spatial resolution at the cortex [37]–[39]. Our recent study suggests that, InI hemodynamic responses can elucidate neuronally related timing information when cross-subject and within-region variability is suppressed by averaging [40].

Several studies have consistently suggested that the sensitivity and stability of Granger causality values can be critically improved if the temporal sampling rate is high enough [26], [41]–[47]. However, to our knowledge, there have been to date no studies empirically demonstrating this. Based on our data showing that the BOLD fMRI signal can reflect neuronal timing at the group level [40], here we hypothesize that increasing the fMRI sampling rate using InI one can provide more robust and sensitive Granger causality estimates compared to conventional multi-slice EPI acquisitions. We test this empirically using InI fMRI with 10-Hz InI sampling rate and a simple visuomotor detection task, which generates feed-forward inter-area information flow [48]. Three different time series were used in this study: fMRI raw time series, hemodynamic response function after General Linear Model, and the estimated neuronal activity using hemodynamic deconvolution. Time series with lower sampling rates (2 Hz, 1 Hz, 0.5 Hz, and 0.2 Hz) were artificially generated by either discarding InI samples or interpolating sub-sampled time series in order to keep the same number of time points. Our results indicate that the high sampling rate provided by InI can robustly improve detection of causal modulations between cortical areas.

## Materials and Methods

### Ethic statement, subjects, and tasks

This study was approved by the Institute Review Boards of National Taiwan University Hospital and National Yang Ming University. Written informed consent approved by the Institute Review Board of National Taiwan University Hospital and National Yang Ming University was obtained from each subject prior to participation. Healthy human volunteers (*n* = 23, 6 females, all right-handed, age 22–30 years) were presented with left or right visual hemifield reversing (8 Hz) checkerboard stimuli in a rapid event-related fMRI design. The hemifield checkerboard subtended a 4.3° visual angle and was generated from 24 evenly distributed radial wedges and eight concentric rings of equal width. The stimuli were presented using the Psychtoolbox [49], [50]. Stimulus duration was 500 ms; the onset of each presentation was randomized with a uniform distribution of inter-stimulus intervals varying from 3 to 16 s (average 10 s). Part of data has been previously reported in studying the correlation between hemodynamic and neuronal activity [51].

The subjects were instructed to press the button upon detecting a visual stimulus, presented randomly at the left or right side of the screen, with the hand ipsilateral to the stimulus. Thus, there were two conditions: right visual hemifield–right hand (R condition) and left visual hemifield–left hand (L condition). This relatively simple task was chosen for its feedforward connectivity from visual to motor systems. Accordingly, our *a priori* hypothesis predicted directional information flow from visual to motor cortices. Twenty-four stimulation epochs were presented during four 240 s runs, resulting in a total of 96 trials per subject.

### Structural MRI acquisitions and reconstructions

Structural *T*_1_-weighted MRIs were acquired with a 3T scanner (Tim Trio, Siemens, Erlangen, Germany) and a 32-channel head phased array coil using a standard MPRAGE sequence (repetition time/echo time/inversion time [TR/TE/TI] = 2,530/3.49/1100 ms, flip angle = 7°, partition thickness = 1.33 mm, image matrix = 256×256, 128 partitions, field-of-view = 21 cm×21 cm). The location of the gray-white matter boundary for each participant was estimated with an automatic segmentation algorithm to yield a triangulated mesh model with approximately 340,000 vertices [52]–[54]. This cortical model was then used to facilitate mapping of the structural image from native anatomical space to a standard cortical surface space [52], [53]. Between-subjects averaging was done by morphing individual data through a spherical coordinate system [55].

### fMRI inverse imaging (InI) acquisitions and reconstructions

BOLD-contrast fMRI data were acquired using inverse imaging [39], [56], which included a reference scan to collect spatial information from different channels in a radio-frequency coil array and a set of dynamic scans to achieve high temporal sampling rate (>10 Hz) with whole brain coverage. The InI reference scan was collected using a single-slice echo-planar imaging (EPI) readout, after exciting one thick coronal slab covering the entire brain (FOV 256 mm×256 mm×256 mm; 64×64×64 image matrix) with the flip angle set to the gray matter Ernst angle of 30° (considering the T1 of gray matter is 1 second at 3T). Partition phase encoding was used to obtain spatial information along the anterior-posterior axis (Y direction). EPI readout had frequency encoding along the superior-inferior (Z direction) and phase encoding along the left-right axis (X direction). We used TR = 100 ms, TE = 30 ms, bandwidth = 2604 Hz and a 12.8-s total acquisition time for the reference scan, allowing the coverage of the whole-brain volume with 64 partitions and two repetitions.

For the InI functional scans, we used the same volume prescription, TR, TE, flip angle, and bandwidth as for the InI reference scan. The principal difference was that, to achieve the high temporal resolution, partition phase encoding (in the Z direction) was removed so that the full volume was excited, and the spins were spatially encoded by a single-slice EPI trajectory, resulting in a coronal X/Z projection image with spatially collapsed projection along the anterior-posterior direction. The *k*-space InI reconstruction algorithm [57] was then used to estimate the spatial information along the anterior-posterior axis. In each run, we collected 2,400 measurements after collecting 32 measurements in order to reach the longitudinal magnetization equilibrium. A total of 4 runs of data were acquired from each participant. The total fMRI acquisition time for each subject was 16 minutes.

InI data were reconstructed time-point by time-point using the minimum-norm estimate method [39], [56], which generated 2,400 reconstructed volumes in each run. Subsequently, we used General Linear Model (GLM) to identify functional areas. The design matrix of GLM included the impulse trains of stimulus onset convolving the finite-impulse-response (FIR) basis function, which included 6-s pre-stimulus baseline and 24-s post-stimulus response, as well as DC and linear drift terms to estimate the hemodynamic responses. For statistical analyses, the noise levels in the reconstructed images were estimated by calculating the standard deviation of the time series during the 6-s pre-stimulus interval. Using these noise estimates, dynamic statistical parametric maps (dSPMs) were derived as the time-point by time-point ratio between the InI reconstruction values and the baseline noise estimates. Such dSPMs can be assumed to be *t* distributed under the null hypothesis of no hemodynamic response [58].

Each subject’s InI time series, including the estimated hemodynamic responses and raw fMRI time series before GLM, were spatially registered into their cortical surface space by using a 12-parameter affine transformation between the volumetric InI reference scan and the MPRAGE anatomical space (FSL, http://www.fmrib.ox.ac.uk/fsl). The resulting spatial transformation was subsequently applied to each time point of the reconstructed InI volume. For inter-subject averaging the individual functional results were co-registered to a spherical brain surface representation [59].

Regions-of-interest (ROIs) were determined by the spatial distribution of the temporally average *t* statistics greater than 4.0 (Bonferroni corrected *p*-value<0.05) between 4 s and 7 s after visual stimulus onset. This interval was chosen to capture the maximum BOLD response. Time courses for each ROI were extracted and averaged within the ROI for each subject separately.

### Echo-planar imaging (EPI) collection and analysis

We also collected EPI with 2 s TR in order to use empirical data to compare the detection sensitivity to causality modulation using InI data. The subjects were instructed to perform the same lateralized visuomotor task as that during the InI acquisitions. Data were collected from 13 subjects over one 4-minute run, where 30 left hemifield and 30 right hemifield visual stimulations were randomly presented. EPI parameters were: TR/TE = 2000/30 ms, field-of-view (FOV) = 220×220 mm, matrix = 64×64, slice thickness = 4 mm, flip angle = 90°. For each subject, thirty-four trans-axial slices with no gap were acquired with the spatial coverage of cerebrum and cerebellum.

EPI fMRI data were first pre-processed for motion correction, slice timing correction, and spatial smoothing (12 mm 3D full-width-half-maximum Gaussian filter) by using the FreeSufer software (http://surfer.nmr.mgh.harvard.edu). We used GLM to identify visual and motor cortices with the FIR bases described above. Estimated hemodynamic activity from each subject was spatially registered to a common surface coordinate system using FreeSufer. The BOLD signals were then averaged across subjects and the dynamic significance of the BOLD signal was calculated using the dSPM procedure described above.

### Time series preparation

In this study, we analyzed the causal modulation using the fMRI time series directly, instead of using the estimated HRF. In preparation of these fMRI time series, GLM was first used to identify the location of visual and sensorimotor cortices (see section **fMRI inverse imaging (InI) acquisitions and reconstructions** and **Echo-planar imaging (EPI) collection and analysis** above). These time series were then averaged within each ROI from each subject. The DC value and the linear drift were also removed from these time series.

This study evaluates how the increased temporal resolution in InI acquisitions and reconstructions can help elucidate the causal interactions in the human visuomotor system. This was done by parametrically downsampling the InI data to generate time series from 0.1-s to 0.5, 1, and 2-s temporal resolution, the last of which is quite common in whole-head fMRI studies. In addition, to avoid the confound of different sampling rates and thus different lengths of time series, we also generated SINC-interpolated time series from the sub-sampled time series such that all time series have the same length in the causality analysis. Finally, we also used EPI time series (before GLM) to estimate causality modulations in order to compare the results based on down-sampled InI data.

### Granger causality and conditional Granger causality analysis

We used an auto-regressive (AR) model to implement the Granger causality modeling. Consider a zero-mean time series at the “destination node”, *y*(*t*). The *p* ^th^-order AR model description of *y*(*t*) is:
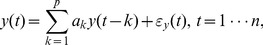
(1)where *a*_k_ are AR model coefficients, and *ε*_y_(*t*) is the residual time series of AR model fitting at the destination node. *n* is the total number of samples in the time series. Provided with the time series from a “source”, *x*(*t*), we can model the bivariate time series of *x*(*t*) and *y*(*t*) as:
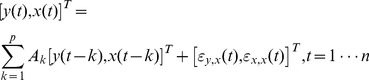
(2)where **A**_k_’s are the AR model coefficient matrices, and [*ε*_y,x_(*t*), *ε*_x,x_(*t*)]*^T^* is the joint bivariate residual time series of AR model fitting at the destination node and source node. The Granger causality metric is
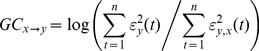
(3)Since the bivariate time series [*y*(*t*), *x*(*t*)]^T^ contains the information of univariate time series *y*(*t*), from Cauchy inequality we can conclude that *G*_x→y_ is well defined and positive since the quotient inside the logarithm is greater or equal to one. Previously, it has been suggested that the inference of *G*_x→y_ can be calculated by referring ((*n*−*p*)/*p*) (exp(*G*_x→y_)–(*n*−2*p*)/(*n*−*p*)) to an *F* distribution with (*p*, *n*−*2p*) degrees of freedom [60].

For each pair of ROI time series (“X” and “Y”), we respectively calculated the Granger causality *G*_x→y_ and *G*_y→x_. Previous simulation studies suggest that naive computation of Granger causality over fMRI signals can be misleading [26]. However, the influence difference (the difference between the pair *G*_x→y_ and *G*_y→x_) can be much more robust [25], [26]. Following this rationale, for each pair of ROIs, we only calculated the difference between two Granger causality values in order to identify the dominant influence direction.

The implementation of the AR modeling was done by the ARFIT algorithm [61], [62], where the optimal model order was jointly determined by model fitting (*i.e.*, favoring the model with smaller power of the residual time series after fitting) and model parsimoniousness (i.e., favoring a lower order model). This implementation has been previously used to explore the causal modulation of epileptic spike propagation measured by MEG [63]. Allowing the AR model ordering ranging between 1 and 20 or the largest order allowed by the number of sample, we used the Bayesian information criterion to choose the optimal order of the AR model. In the Granger causality analysis on time series of a pair of ROIs, we used the minimum of the estimated AR model orders to estimate the GC for the sake of model simplicity.

We used a non-parametric approach to estimate the statistical significance of the influence difference, because the null distribution of such influence difference has no analytic form. Furthermore, if we want to use the F/Chi-square distributions in causality estimation, samples in the time series need to be independent. This may not be the case in BOLD-contrast fMRI time series with different sampling rates. Our non-parametric analysis started from generating the null distribution of influence difference using the Adjusted Amplitude Fourier Transform (AAFT), a method of generating surrogate time series while preserving the linear correlation structure of the original time series and the marginal distribution [64]. The permutation procedure was repeated 1000 times for Granger causality analysis and 100 times for conditional Granger causality analysis, because the latter analysis took a longer computation time. We defined the *p*-value as the number of occurrences of the Granger causality values using a swapped source time series exceeding the Granger causality value using the original source time series. The *p*-value of the Granger causality difference in the group analysis was calculated accordingly as the ratio between the number of the Granger causality difference values from permuted time series higher than that from the original time series in each subject and the total number of permutation test across subjects. Note that such a *p*-value calculation amounts to a fixed-effect analysis.

Granger causality can be complicated by a “common source” problem. Specifically, the causal modulation between a pair of time series can be mediated through other time series within the set of analyzed time series. This problem has been partially addressed by the conditional Granger causality approach [25], [65]–[67]. In the case of studying the causal modulations between the time series pair [*y*(*t*), *x*(*t*)]^T^, suppose that a multivariate auto-regressive model can be used to describe all other time series [*z*(*t*)]^T^. The conditional Granger causality c*G*_x→y_ is calculated as the ratio between the residual variance in the joint time series [*y*(*t*), *z*(*t*)]^T^ and the residual variance in the joint time series [*x*(*t*), *y*(*t*), *z*(*t*)]^T^:
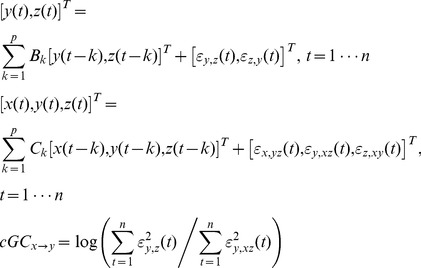
(4)Heuristically, conditional Granger causality evaluates the improvement of the additionally explained variance in the target time series by adding the source time series after controlling the information in all other time series. Statistical significance can be similarly derived from the empirical null distribution.

All calculations were done using Matlab (Mathworks, Natick, MA, USA).

## Results

**Figure 1:**
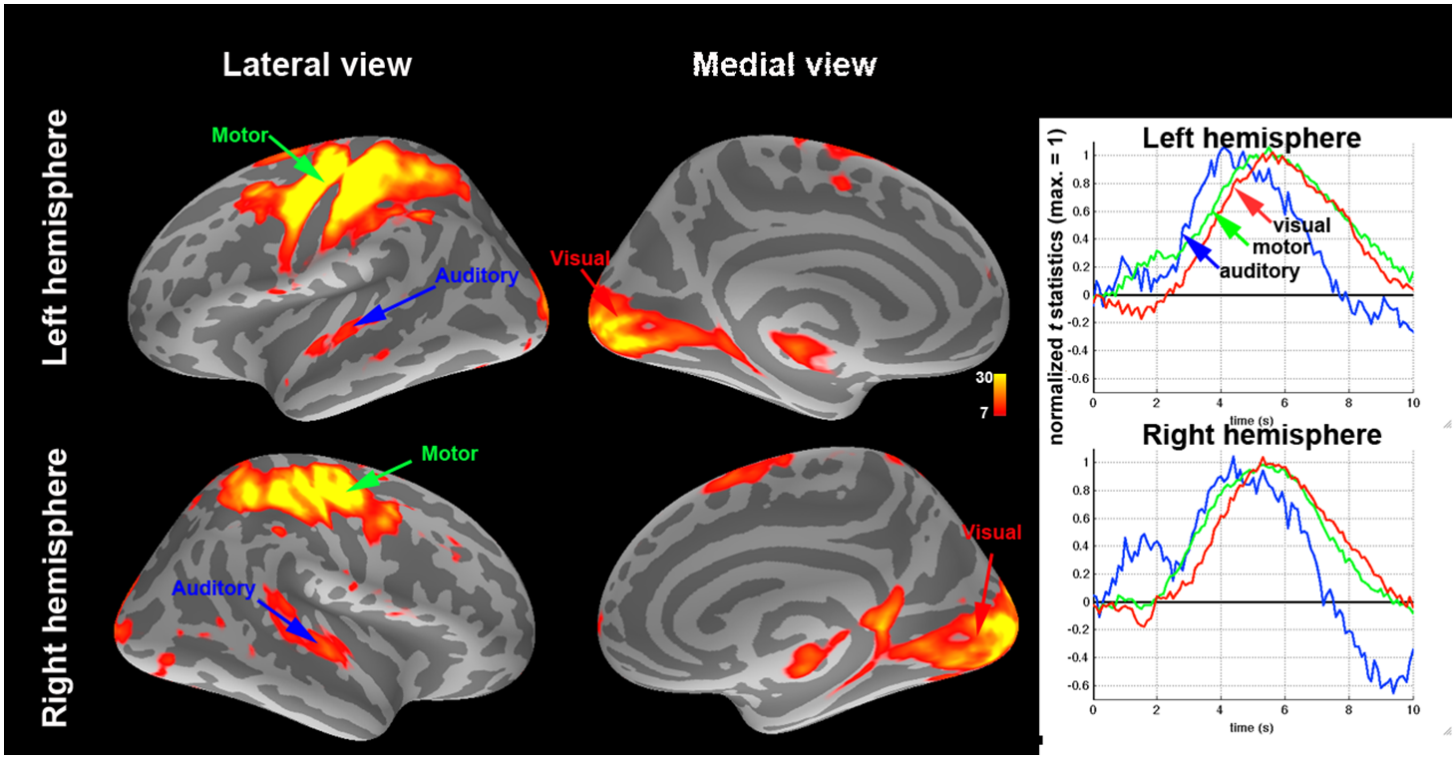
(Left) Locations of the five ROIs (*t* statistics of the BOLD signal averaged between 4.0 s and 7.0 s after the visual stimulus onset) in each hemisphere. (Right) Hemodynamic time courses and estimated neuronal activity using hemodynamic deconvolution at five ROIs.

The visuomotor task elicited strongest BOLD signal at the left hemisphere, contralateral to the visual stimulus/motor response. Thus, results for the L condition are reported in the right hemisphere and the R condition in the left hemisphere. We identified five regions-of-interest (ROIs), including visual cortex (V), parietal cortex (PPC), pre-motor cortex (PreM), somatosensory cortex (S), and motor cortex (M) for both L and R conditions, as shown in **Figure 1**. Notably, the details of the average time courses (**Figure 1**) show sequential BOLD activity with different onsets, time-to-half (TTH), time-to-peak (TTP) timing, and width of regional hemodynamic response (**Table 1**).

**Table 1:** Timing indices and the full-width-half-maximum (FHWM) of the group-average hemodynamic responses in five regions-of-interest.

		V	PCC	PreM	S	M
**Onset (s)**	**L**	0.63	0.91	0.69	1.04	1.18
	**R**	0.60	1.03	0.84	1.04	1.19
**Time-to-half (s)**	**L**	1.70	1.90	1.90	2.20	2.40
	**R**	1.70	2.00	2.20	2.20	2.40
**Time-to-peak (s)**	**L**	3.60	3.70	4.00	4.10	4.50
	**R**	3.70	4.10	4.20	4.20	4.40
**FWHM (s)**	**L**	4.60	4.30	5.10	4.60	4.70
	**R**	4.80	4.80	4.70	4.60	4.60

V: visual cortex; PCC: parietal cortex; PreM: pre-motor cortex; S: sensorimotor cortex; M: motor cortex. L: left hemisphere (R condition). R: right hemisphere (L condition).

**Figure 2:**
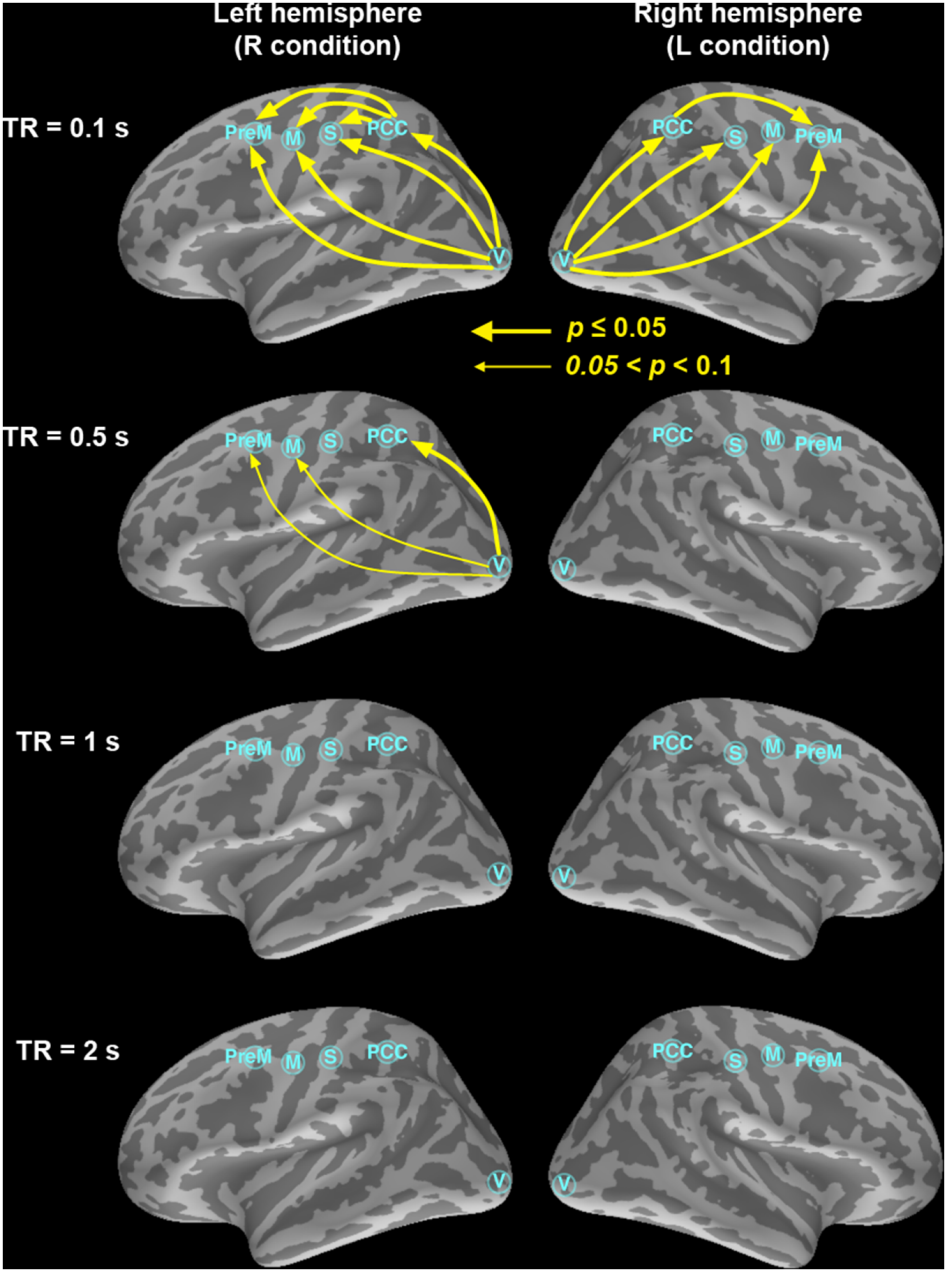
The dominant information flow calculated from the difference between two uni-directional Granger estimates among the visual (V), PPC, premotor (PreM), somatosensory (S), and motor (M) cortex ROIs at TR = 0.1 s, 0.5 s, 1 s, and 2 s. Significant causal modulations (*p*≤0.05) were shown in thick yellow arrows and connections showing a trend of causal modulation (0.05<*p*≤0.1) were shown in thin yellow arrows.

**Figure 2** **.** shows that increasing the fMRI sampling rate significantly improved the sensitivity of our Granger causality estimates, as indicated by the emergence of multiple directional influences consistent with the visuomotor task. The dominant direction of information flow between any two ROIs (denoted X and Y) was determined by calculating the difference (G_X→Y_–G_Y→X_) between the two uni-directional Granger causality estimates [23], [26]. **Table 2** lists the order of the optimal AR model for each ROI time series. *P*-values of all directions of information flow between ROI’s were listed in **Table 3**. At the highest temporal resolution (TR = 0.1 s), significant bottom-up causal influences were observed from visual to PPC, premotor, somatosensory, and motor cortices in both left and right hemispheres. Reducing the temporal resolution clearly decreased the number of significant causality influences. At TR = 0.5 s, left hemisphere still shows three strong feedforward connections. However, the significance levels of two connections (from visual cortex to premotor and motor cortices) have decreased. Further lowering the sampling rate down to 1 s and 2 s, the conventional TR for whole-head EPI, shows no significant feedforward connections. In addition to the influence differences, we also report the Granger causality values and their associated significance in **Table 4**, showing that almost all causality estimates were significant at TR< = 1 s and non-significant at TR = 2 s and that the majority of statistically significant GC connections were bidirectional.

**Table 2:** The optimal order of the AR model of the time series at five ROI’s.

		V	PCC	PreM	S	M
**TR = 0.1 s**	**L**	11	11	11	11	12
	**R**	10	8	9	9	9
**TR = 0.5 s**	**L**	3	3	3	3	3
	**R**	4	3	4	4	4
**TR = 1 s**	**L**	1	1	2	1	1
	**R**	2	2	2	2	2
**TR = 2 s**	**L**	1	1	1	1	1
	**R**	1	1	1	1	1

V: visual cortex; PCC: parietal cortex; PreM: pre-motor cortex; S: sensorimotor cortex; M: motor cortex. L: left hemisphere (R condition). R: right hemisphere (L condition).

**Table 3:** *P*-values of all directions of information flow estimated by Granger causality between ROI’s at different sampling rates from 1000 bootstrap iterations.

TR = 0.1 s		FROM				
		V	PPC	PreM	S	M
TO	V	N/A	1-*p*; 1-*p*	1-*p*; 1-*p*	1-*p*; 1-*p*	1-*p*; 1-*p*
	PPC	<0.001; 0.006	N/A	1-*p*; 1-*p*	1-*p*; 1-*p*	1-*p*; 1-*p*
	PreM	<0.001; <0.001	<0.001; 0.006	N/A	1-*p*; 1-*p*	1-*p*; 1-*p*
	S	<0.001; 0.016	0.002; 0.576	0.883; 0.897	N/A	1-*p*; 1-*p*
	M	<0.001; 0.003	<0.001; 0.390	0.110; 0.861	0.904; 0.256	N/A
**TR = 0.5 s**		**FROM**				
		**V**	**PPC**	**PreM**	**S**	**M**
TO	V	N/A	1-*p*; 1-*p*	1-*p*; 1-*p*	1-*p*; 1-*p*	1-*p*; 1-*p*
	PPC	0.027; 0.573	N/A	1-*p*; 1-*p*	1-*p*; 1-*p*	1-*p*; 1-*p*
	PreM	0.069; 0.259	0.305; 0.618	N/A	1-*p*; 1-*p*	1-*p*; 1-*p*
	S	0.120; 0.358	0.597; 0.152	0.891; 0.416	N/A	1-*p*; 1-*p*
	M	0.076; 0.541	0.378; 0.128	0.489; 0.387	0.304; 0.470	N/A
**TR = 1 s**		**FROM**				
		**V**	**PPC**	**PreM**	**S**	**M**
TO	V	N/A	1-*p*; 1-*p*	1-*p*; 1-*p*	1-*p*; 1-*p*	1-*p*; 1-*p*
	PPC	0.377; 0.542	N/A	1-*p*; 1-*p*	1-*p*; 1-*p*	1-*p*; 1-*p*
	PreM	0.119; 0.648	0.339; 0.540	N/A	1-*p*; 1-*p*	1-*p*; 1-*p*
	S	0.224; 0.442	0.263; 0.324	0.551; 0.518	N/A	1-*p*; 1-*p*
	M	0.206; 0.381	0.268; 0.280	0.374; 0.419	0.402; 0.426	N/A
**TR = 2 s**		**FROM**				
		**V**	**PPC**	**PreM**	**S**	**M**
TO	V	N/A	1-*p*; 1-*p*	1-*p*; 1-*p*	1-*p*; 1-*p*	1-*p*; 1-*p*
	PPC	0.744; 0.280	N/A	1-*p*; 1-*p*	1-*p*; 1-*p*	1-*p*; 1-*p*
	PreM	0.669; 0.806	0.463; 0.833	N/A	1-*p*; 1-*p*	1-*p*; 1-*p*
	S	0.713; 0.338	0.313; 0.610	0.636; 0.381	N/A	1-*p*; 1-*p*
	M	0.668; 0.356	0.252; 0.543	0.602; 0.396	0.389; 0.382	N/A

Each cell contains two *p*-values. The left *p*-value is for the left hemisphere (R condition) and the right *p*-value is for the right hemisphere (L condition). Only lower-triangular off-diagonal entries are listed. Upper-triangular off-diagonal entries can be calculated by (1–listed *p*-value). V: visual cortex; PCC: parietal cortex; PreM: pre-motor cortex; S: sensorimotor cortex; M: motor cortex. L: left hemisphere (R condition). R: right hemisphere (L condition).

**Table 4:** *P*-values of Granger causality values between ROI’s at different sampling rates.

TR = 0.1 s		FROM							
		V	PPC			PreM		S	M
TO	V	N/A	<0.001; <0.001			<0.001; <0.001		<0.001; <0.001	<0.001; <0.001
	PPC	<0.001; <0.001	N/A			<0.001; <0.001		<0.001; <0.001	<0.001; <0.001
	PreM	<0.001; <0.001	<0.001; <0.001			N/A		<0.001; <0.001	<0.001; <0.001
	S	<0.001; <0.001	<0.001; <0.001			<0.001; <0.001		N/A	<0.001; <0.001
	M	<0.001; <0.001	<0.001; <0.001			<0.001; <0.001		<0.001; <0.001	N/A
**TR = 0.5 s**		**FROM**							
		**V**	**PPC**			**PreM**	**S**		**M**
TO	V	N/A	0.017; <0.001			0.008; <0.001	<0.001; <0.001		<0.001; <0.001
	PPC	<0.001; <0.001	N/A			0.004; 0.002	<0.001; <0.001		0.001; <0.001
	PreM	<0.001; <0.001	0.007; <0.001			N/A	0.002; <0.001		0.002; <0.001
	S	<0.001; <0.001	0.002; <0.001			0.008; 0.002	N/A		0.007; <0.001
	M	<0.001; <0.001	0.001; <0.001			0.004; <0.001	0.006; <0.001		N/A
**TR = 1 s**		**FROM**							
		**V**	**PPC**	**PreM**				**S**	**M**
TO	V	N/A	0.067; 0.047	0.090; 0.070				0.039; 0.038	0.047; 0.031
	PPC	0.044; 0.023	N/A	0.133; 0.052				0.201; 0.020	0.100; 0.030
	PreM	0.395; 0.036	0.107; 0.036	N/A				0.201; 0.038	0.050; 0.052
	S	0.040; 0.030	0.076; 0.009	0.182; 0.026				N/A	0.158; 0.004
	M	0.010; 0.022	0.021; 0.029	0.054; 0.033				0.147; 0.058	N/A
**TR = 2 s**		**FROM**							
		**V**	**PPC**		**PreM**			**S**	**M**
TO	V	N/A	0.240; 0.170		0.267; 0.085			0.246; 0.196	0.195; 0.151
	PPC	0.167; 0.113	N/A		0.205; 0.172			0.356; 0.175	0.221; 0.132
	PreM	0.298; 0.172	0.172; 0.237		N/A			0.199; 0.235	0.188; 0.179
	S	0.238; 0.124	0.222; 0.136		0.282; 0.190			N/A	0.164; 0.167
	M	0.140; 0.118	0.179; 0.157		0.318; 0.200			0.169; 0.192	N/A

Each cell contains two *p*-values. The left *p*-value is for the left hemisphere (R condition) and the right *p*-value is for the right hemisphere (L condition). V: visual cortex; PCC: parietal cortex; PreM: pre-motor cortex; S: sensorimotor cortex; M: motor cortex. L: left hemisphere (R condition). R: right hemisphere (L condition).

Granger estimates can be confounded by one source driving multiple targets. To reduce confounds related to potential common sources, we also calculated the difference conditional Granger causality values among pairs of the time series in 5 ROIs. Different from the Granger causality, conditional Granger causality estimates the specific information flow between two chosen ROIs while the information from all other ROIs through direct or indirect information flow are removed [65], [67], [68]. *P*-values are marginally different between Granger causality analysis and conditional Granger causality analysis in all sampling rates (**Table 5**). Again, increasing the TR gradually reduced the number of significant paths – no significant causal modulation was observed in both hemispheres at TR = 1 s and 2 s.

**Table 5:** *P*-values of all directions of information flow estimated by conditional Granger causality between ROI’s at different sampling rates from 100 bootstrap iterations.

TR = 0.1 s		FROM				
		V	PPC	PreM	S	M
TO	V	N/A	1-*p*; 1-*p*	1-*p*; 1-*p*	1-*p*; 1-*p*	1-*p*; 1-*p*
	PPC	<0.01; 0.01	N/A	1-*p*; 1-*p*	1-*p*; 1-*p*	1-*p*; 1-*p*
	PreM	<0.01; <0.01	<0.01; 0.01	N/A	1-*p*; 1-*p*	1-*p*; 1-*p*
	S	<0.01; <0.01	<0.01; 0.57	0.88; 0.89	N/A	1-*p*; 1-*p*
	M	<0.01; <0.01	<0.01; 0.37	0.11; 0.87	0.89; 0.26	N/A
**TR = 0.5 s**		**FROM**				
		**V**	**PPC**	**PreM**	**S**	**M**
TO	V	N/A	1-*p*; 1-*p*	1-*p*; 1-*p*	1-*p*; 1-*p*	1-*p*; 1-*p*
	PPC	0.03; 0.54	N/A	1-*p*; 1-*p*	1-*p*; 1-*p*	1-*p*; 1-*p*
	PreM	0.07; 0.28	0.30; 0.62	N/A	1-*p*; 1-*p*	1-*p*; 1-*p*
	S	0.10; 0.38	0.69; 0.15	0.88; 0.40	N/A	1-*p*; 1-*p*
	M	0.08; 0.57	0.41; 0.13	0.49; 0.38	0.22; 0.48	N/A
**TR = 1 s**		**FROM**				
		**V**	**PPC**	**PreM**	**S**	**M**
TO	V	N/A	1-*p*; 1-*p*	1-*p*; 1-*p*	1-*p*; 1-*p*	1-*p*; 1-*p*
	PPC	0.37; 0.57	N/A	1-*p*; 1-*p*	1-*p*; 1-*p*	1-*p*; 1-*p*
	PreM	0.13; 0.64	0.35; 0.54	N/A	1-*p*; 1-*p*	1-*p*; 1-*p*
	S	0.20; 0.50	0.27; 0.32	0.56; 0.49	N/A	1-*p*; 1-*p*
	M	0.22; 0.45	0.29; 0.29	0.36; 0.40	0.38; 0.43	N/A
**TR = 2 s**		**FROM**				
		**V**	**PPC**	**PreM**	**S**	**M**
TO	V	N/A	1-*p*; 1-*p*	1-*p*; 1-*p*	1-*p*; 1-*p*	1-*p*; 1-*p*
	PPC	0.73; 0.25	N/A	1-*p*; 1-*p*	1-*p*; 1-*p*	1-*p*; 1-*p*
	PreM	0.67; 0.78	0.49; 0.84	N/A	1-*p*; 1-*p*	1-*p*; 1-*p*
	S	0.70; 0.33	0.34; 0.64	0.66; 0.39	N/A	1-*p*; 1-*p*
	M	0.65; 0.39	0.27; 0.56	0.58; 0.38	0.37; 0.39	N/A

Each cell contains two *p*-values. The left *p*-value is for the left hemisphere (R condition) and the right *p*-value is for the right hemisphere (L condition). Only lower-triangular off-diagonal entries are listed. Upper-triangular off-diagonal entries can be calculated by (1–listed *p*-value). V: visual cortex; PCC: parietal cortex; PreM: pre-motor cortex; S: sensorimotor cortex; M: motor cortex. L: left hemisphere (R condition). R: right hemisphere (L condition).

**Figure 3:**
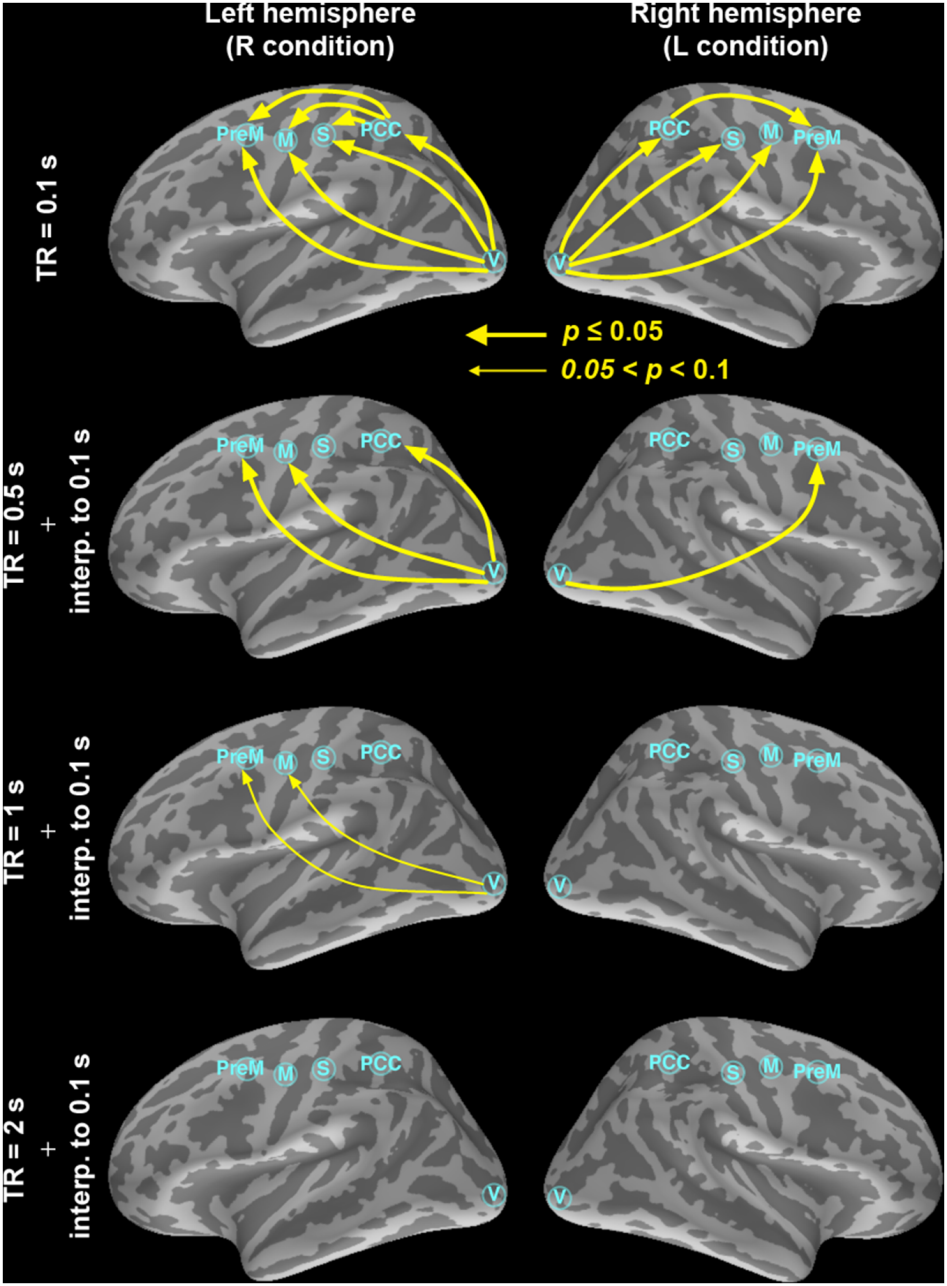
The dominant information flow calculated from the difference between two uni-directional Granger estimates among the visual (V), PPC, premotor (PreM), somatosensory (S), and motor (M) cortex ROIs at TR = 0.1 s, 0.5 s, 1 s, and 2 s. The time series at 0.5 s, 1 s, and 2 s were SINC interpolated such that all time series are of the same length as the 0.1 s time series, which contains the real measured data. Significant causal modulations (*p*≤0.05) were shown in thick yellow arrows and connections showing a trend of causal modulation (0.05<*p*≤0.1) were shown in thin yellow arrows. Importantly, only very little improvement is observed in the estimates where the number of observations is artificially increased using the SINC interpolation procedure: the strongest connectivity patterns are, clearly, observed in the 0.1-s condition with only real data.

To confirm that the increased sensitivity in detecting feedforward connections is not due to different time series length, we SINC interpolated the time series from the subsampled data such that all time series had the same length before Granger causality analysis. For example, TR = 0.5 s data were first down-sampled by 5-fold from the densely sampled time series with TR = 0.1 s and subsequently SINC interpolated by 5-fold. Results in **Figure 3** shows that, while interpolation can artificially help improve the detection, slow sampling rates at the order of 1 s cannot provide significant feedforward connection estimates in left and right hemispheres. Specifically, we found that the significance levels for the V→M and V→PreM connections in the left hemisphere decreased (increased *p*-value) at TR = 0.5 s. A significant V→PreM connection shows up at TR = 0.5 s in the right hemisphere after data interpolation. Results with TR = 1 s suggested possible V→PreM and V→M connections. However, the trend of losing detecting feedforward connections at a slower sampling rate prevails. In particularly, TR = 2 s, the typical whole-head EPI sampling rate, shows no significant connections in both hemispheres.

Finally, in addition to using down-sampled InI data, we also used EPI data with TR = 2 s record the subjects’s BOLD signal at visual and motor cortices in the same lateralized visuomotor task. Consistent with the down-sampled InI simulations (**Table 3**), we found that there was no significant V→M causal modulation in both R and L conditions (*p*-values = 0.27 and 0.22 for R and L conditions, respectively).

## Discussion

Our results demonstrate that the inverse imaging method with a 20-fold increase in fMRI temporal resolution can greatly improve the detection power of causality analysis in the human brain. Using whole-head InI acquisitions with 100 ms temporal resolution, we were able to infer directional causal influences between five functional areas activated during a lateralized visuomotor task in each hemisphere with both Granger causality and conditional Granger causality analyses. Consistent with the *a priori* predicted pattern of functional connectivity in a visuomotor choice-reaction task [48], the results were dominated by feedforward influences from visual to sensorimotor, premotor, and posterior parietal regions. These observations strongly underline the importance of high temporal sampling rates in determining effective connectivity, because the connectivity patterns only emerged at the 100 ms TR (**Figures 2** **and** **3**). Controlling the number of time points by interpolating the time series with a lower sampling rate can only partially mitigate the problem of losing sensitivity in correct causality estimates (**Figure 3**).

The advantage of higher temporal resolution has been suggested by simulation studies [26], [41]–[46], but has not been previously shown with empirical data. Moreover, the previous simulation studies assumed that regular EPI would be used, where any increase in fMRI temporal resolution has to be traded off for smaller spatial coverage, poorer spatial resolution, or jittered designs that greatly increase the duration of the imaging session [2], [69]. Specifically, limited by the gradient slew rate, the acquisition of each echo-planar imaging slice takes approximately 80 ms at ∼3 mm×3 mm spatial resolution. Assuming that 30 slices would be needed to cover the entire brain, a jittered stimulus design would require a 30-fold increase in data acquisition time, leading to impractical session durations (several hours). With the InI approach in the present study, we obtained a whole-head volume in 100 ms, but it is possible to speed up the acquisition even further. The temporal resolution of InI is determined by TE (30 ms) optimized for the BOLD contrast and desired field-of-view/spatial resolution. Using partial Fourier acquisition and a higher readout bandwidth, the readout time could be reduced from 32 ms (2 KHz bandwidth and 64 lines) to 16 ms (6/8 partial Fourier, 48 lines at 3 KHz bandwidth) at the cost of reduced SNR. However, if using an echo-shifting pulse sequence [56], [70], it is possible to achieve 20 ms TR at 30 ms TE. These future developments could allow for testing causal modulations between hemodynamic time series with putative latency differences well below 100 ms.

In this study we chose InI for its whole-brain coverage, reasonable spatial resolution at cortex, and high sampling rate (TR = 0.1 s). Note that recently there are other fast fMRI acquisition methods, such as generalized InI [71], fast fMRI [72], MR-encephalography (MREG) [73], and simultaneous multi-slice EPI [74] methods. Each method has different magnetization excitation strategies and image reconstruction algorithms. Yet all of them can provide a sub-second sampling rate. As we demonstrate that fast fMRI acquisitions can help improve detecting causal modulations in human brain in general, researchers can choose any method adaptively under different theoretical or practical concerns without losing the detection power.

InI trades off a small amount of spatial resolution in one encoding direction for a substantially higher temporal resolution. Depending on the reconstruction methods, InI has approximately 5 mm spatial resolution at cortex using a 32-channel head coil array at 3T [37]–[39], which is in the range of spatial filtering/smoothing that is typically applied in echo-planar imaging as a post-processing step. Without reaching the limit based on the electromagnetic theory [75], [76], using a head coil array of more channels at a higher field (for example, 7T) could further improve the spatial resolution of InI. Novel reconstruction methods targeted at suppressing the point spread function, such as using the minimum L-1 norm constraint [77], can also be used to improve the spatial resolution in studies where the highest possible spatial resolution is critical.

In addition to the neurophysiologically expected feedforward modulation from V→PPC→preM→M, our InI results also suggest direct feed-forward connectivity from V to preM, M, and S. Although these influences can be supported by animal models showing direct structural connections from visual to sensory and motor cortices in rats [78], it is also possible that different neurovascular coupling in the different ROIs caused smearing of neuronal temporal information that reduced the sensitivity of GC to detect the dominant feedforward connections in our estimates.

In contrast to the present finding, previous studies have shown that EPI data may show causal modulation in the human sensorimotor system using EPI with TR in the order of 2 s [24], [26]. However, this seeming discrepancy may be explained by the different nature of the present two-choice reaction-time task and those used in many previous studies. Here, the group average reaction time was less than 400 ms [40]. Therefore, without considering the differential vascular responses at visual and sensorimotor cortices, the BOLD signal time series in the visual and sensorimotor cortices were expected to be delayed by only a few hundreds of milliseconds. Such a latency is difficult to be resolved by regular EPI with TR = 2 s. Consequently, one can expect that quite similar BOLD signal time series in visual and sensorimotor cortices were elicited during this task. Two similar time series are actually difficult to use Granger causality analysis to reveal any causal modulation between them. In a simple theoretical example with two *identical* time series, it is clear that the residual time series in modeling one time series will *not* be further improved by providing the other time series, because all information has been provided by the first time series already. In such a case, the Granger causality value will be insignificant because *no* further reduction on the power of residual time series after modeling the first one.

**Figure 4:**
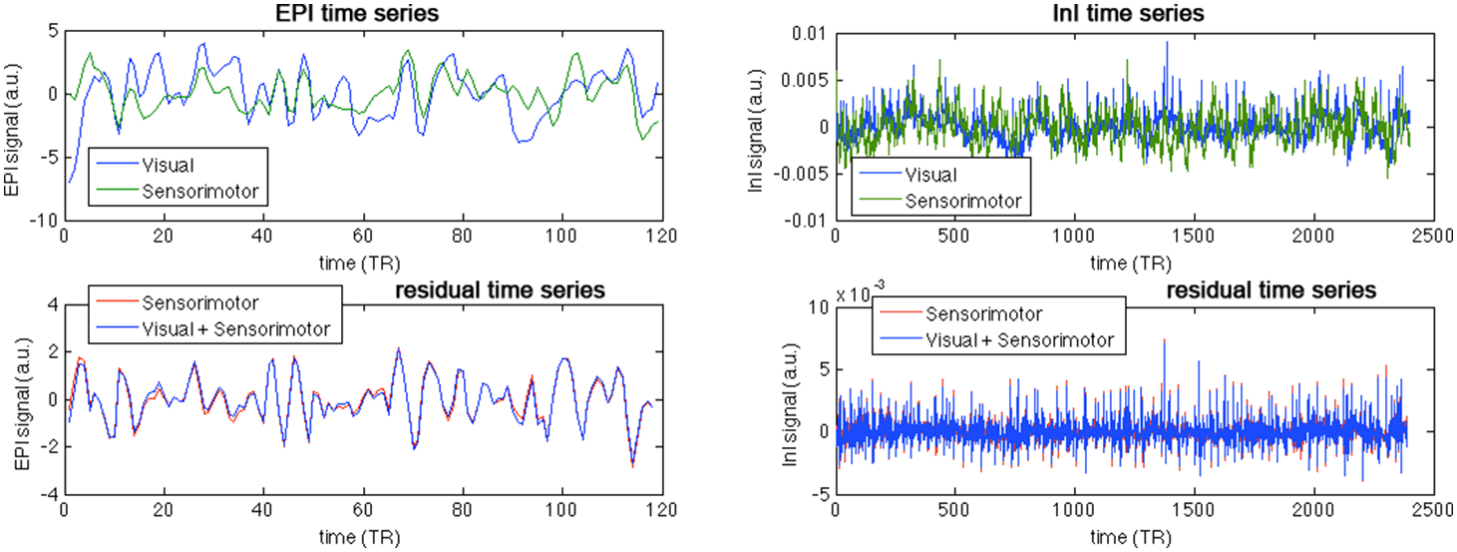
The EPI (left column) and InI (right column) time series at the visual and sensorimotor cortices from a representative subject (top panel) and the residual EPI (left column) and InI (right column) time series at the sensorimotor cortex after AR modeling using the sensorimotor cortex time series alone and both sensorimotor and visual cortices (bottom panel).

To support our argument above, **Figure 4** shows an example of two highly correlating (*r* = 0.446) EPI time series at visual and sensorimotor cortices from one representative subject. **Figure 4** also shows the residual time series after modeling the sensorimotor time series when either only the sensorimotor cortex time series was provided, or both the sensorimotor and visual cortices time series were provided. The residual time series power only changed marginally (108.0→105.6; 2.3% reduction), in line with our main result of no significant Granger causality modulation between visual and sensorimotor cortices as measured by EPI with TR = 2 s. For comparison, we also show the InI time series in visual and sensorimotor cortices from one representative subject, and the residual time series after modeling the sensorimotor time series when either only the sensorimotor cortex time series was provided, or both the sensorimotor and visual cortices time series were provided. Visually, it is difficult to discern the precedence of either time series. However, numerically the variance of residual time series at the sensorimotor cortex was apparently reduced when the visual cortex time series was provided (0.0026→0.0023; 11.5% reduction).

Finally, it should be noted that BOLD-contrast fMRI measures the vascular responses secondary to neuronal events. On top of information reflecting neuronal activity, there are well documented vascular confounds in fMRI time series [34], [79]. Our previous study has shown that, with group averaging and improved sampling rate, inter-regional hemodynamic responses can be significantly correlated with inter-regional neuronal activity [51]. In this study, we chose a relatively simple two-choice reaction-time task with *a priori* assumption of observing strong feed-forward connectivity from the visual to the motor cortices. Such findings are consistent with a previous study [80] suggesting that fast sampling can eliminate confounding effects of differential HRF delays over areas by fine features of the HRF waveform (see also **Table 1**). However, while our results suggest that it is feasible to increase the fMRI sampling rate to enhance sensitive of causality estimates, caution must be always exercised when interpreting the results provided by these methods in the context of tasks eliciting more complex feed-forward/feedback information flow patterns.

Taken together, our results suggest that using MR InI with a 100 ms sampling interval (20-fold faster than conventional EPI), the sensitivity of detecting causal connectivity is significantly improved. We expect that this method can be used in other fMRI experiments to reveal effective connectivity when the vascular confound of the BOLD-contrast fMRI is carefully controlled and thus potentially open up entirely new possibilities for non-invasive imaging of effective connectivity in the human brain.
