# Correction: Increasing fMRI Sampling Rate Improves Granger Causality Estimates

**DOI:** 10.1371/journal.pone.0109064

**Published:** 2014-09-23

**Authors:** The PLOS ONE Staff

Figure 1 is incorrect. The authors have provided a corrected version here.

**Figure 1:**
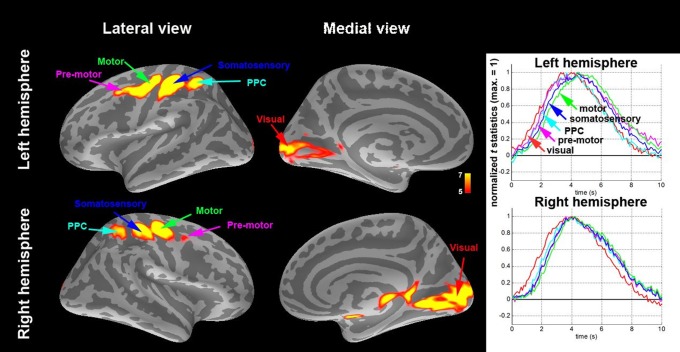
(Left) Locations of the five ROIs (t statistics of the BOLD signal averaged between 4.0 s and 7.0 s after the visual stimulus onset) in each hemisphere. (Right) Hemodynamic time courses and estimated neuronal activity using hemodynamic deconvolution at five ROIs.
